# Risk assessment of resistance to diflubenzuron in *Musca domestica*: Realized heritability and cross-resistance to fourteen insecticides from different classes

**DOI:** 10.1371/journal.pone.0268261

**Published:** 2022-05-13

**Authors:** Abdulwahab M. Hafez

**Affiliations:** Department of Plant Protection, College of Food and Agriculture Sciences, Pesticides and Environmental Toxicology Laboratory, King Saud University, Riyadh, Saudi Arabia; University of Idaho, UNITED STATES

## Abstract

The *Musca domestica* L. is a well-known vector for a number of livestock and human diseases. One major challenge for maintaining effective control of this pest is its propensity to develop resistance to insecticides. This study utilized laboratory selection and realized heritability methods to examine the risk of resistance development to diflubenzuron in *Musca domestica* L. Cross-resistance (CR) to fourteen other insecticides was measured in diflubenzuron-selected (Diflu-SEL) strain which was selected for 20 generations. The resistance ratio (RR) of Diflu-SEL larvae to diflubenzuron increased from 30.33 in generation five (G_5_) to 182.33 in G_24_ compared with the susceptible strain, while realized heritability (*h*^2^) was 0.08. The number of needed generations (G) for a tenfold increase in the median lethal concentration (LC_50_) for diflubenzuron ranged from 4 to 45 at *h*^2^ values of 0.08, 0.18, and 0.28, at a slope of 1.51. At *h*^2^ = 0.08 and slopes of 1.51, 2.51, and 3.51, the number of needed G for a tenfold increase in the LC_50_ ranged from 9 to 104. The level of CR shown by the Diflu-SEL strain to all other fourteen tested insecticides (insect growth regulators, organophosphates, and pyrethroids) was either absent or very low compared to the field population. The value of *h*^2^ and the absent or low CR indicate potential successful management of resistance to diflubenzuron and recommend the use of the tested insecticides in rotation with diflubenzuron to control *M*. *domestica*.

## Introduction

The house fly, *Musca domestica* L. (Diptera: Muscidae), is a worldwide insect pest to livestock, and has the potential to act as a vector for a number of livestock and human diseases, including diarrheal diseases and avian influenza [1–3]. This pest breeds rapidly in and near homes in discarded waste and in animal manure from livestock facilities [4]. The removal of animal manure at livestock facilities accompanied by an integrated program of chemical insecticides are necessary for the satisfactory control of *M*. *domestica* [5].

Insecticides based on insect growth regulators (IGRs) include juvenile hormone mimics, ecdysone agonists, and chitin synthesis inhibitors. IGRs disrupt metamorphosis so that the insects do not develop into adults or developed adults have a significantly reduced reproductive rate [6,7]. These insecticides, considered environmentally friendly, are potent larvicides for controlling insect vectors, including *M*. *domestica*, worldwide [8–13]. Diflubenzuron, an IGR, is a chitin synthesis inhibitor that disrupts cuticle formation and is one of the most effective larvicides for controlling different insect pests, including *M*. *domestica* [12,14–16]. However, striking diflubenzuron resistance has now been documented in a number of insect pests, including *M*. *domestica* [9,17], *Culex pipiens* L. (Diptera: Culicidae) [18], *Lucilia cuprina* (Wiedemann) (Diptera: Calliphoridae) [19,20], and *Bovicola ovis* (Schrank) (Phthiraptera: Trichodectidae) [21].

Assessment of the risk of insecticide resistance provides valuable data for proactively devising or improving resistance management programs through which susceptibility can be maintained [22–24]. The risk of resistance development to insecticides can be assessed by laboratory selection and by measuring realized heritability values [25,26]. Previous studies have documented these parameters in *M*. *domestica* for many insecticides, including lambda-cyhalothrin [27], methoxyfenozide [28], pyriproxyfen [23], fipronil [3], emamectin benzoate [29], cyromazine [30], and flonicamid [24].

Analysis of the potential for cross-resistance (CR) to insecticides is important for defining their efficiency and for informing programs of rotational usage of potent insecticides to limit resistance problems in pest populations [1,31]. Patterns of CR to insecticides having similar or different modes of action have been documented in *M*. *domestica* strains [1,17,24,31–35]. However, CR potential in diflubenzuron-resistant *M*. *domestica* in Saudi Arabia is still unexplored. The aims of the current study were to explore the pattern of CR to fourteen insecticides in diflubenzuron-selected *M*. *domestica*, to measure the risk of resistance to diflubenzuron through laboratory selection, and to measure realized heritability values.

## Materials and methods

### Ethics approval

The *M*. *domestica* population was collected from the dairy farm based on a personal communication with the owner and no specific permit was required.

### Insecticides

Fifteen formulated insecticides from three classes (IGR, organophosphate, and pyrethroid) were used in the bioassays (Table 1).

**Table 1:** The list of tested insecticides.

Insecticide class	Active ingredient	Trade name	Company	Formulation	IRAC Mode of action
Insect Growth Regulators	Diflubenzuron	Diflon	Saudi Delta Company, Saudi Arabia	250WP	Inhibitors of chitin biosynthesis affecting CHS1
	Triflumuron	Starycide	Bayer Crop Sciences, Germany	480SC	
	Pyriproxyfen	Admiral	Sumitomo Chemicals, Japan	10EC	Juvenile hormone mimics
	Methoxyfenozide	Runner	Dow Agrosciences, United Kingdom	24SC	Ecdysone receptor agonists
	Cyromazine	Novasat	Astranova Chemicals, Saudi Arabia	75WP	Molting disruptors, Dipteran
Organophosphate	Fenitrothion	Fentox	Pioneers Chemicals Factory Co., Saudi Arabia	500EC	Acetylcholinesterase (AChE) inhibitors
	Malathion	Delthion	Saudi Delta Company, Saudi Arabia	570EC	
	Diazinon	Diazinon	APCO, Saudi Arabia	60EC	
	Pirimiphos-methyl	Actikil	Astrachem, Saudi Arabia	500EC	
	Chlorpyrifos	Chlorfet	Masani Chemicals, Jordan	48EC	
Pyrethroid	Alpha-cypermethrin	Alphaquest	Astrachem, Saudi Arabia	100EC	Sodium channel modulators
	Bifenthrin	Biflex	FMC, Belgium	8SC	
	Deltamethrin	K-Othrine	Bayer Crop Sciences, France	25SC	
	Cyfluthrin	Solfac	Bayer Crop Sciences, Germany	050EW	
	Cypermethrin	Montothrin	Montajat Veterinary Tool Products, Saudi Arabia	10EC	

### *Musca domestica* strains

Between 150 and 200 *M*. *domestica* mixed sexes adults were captured in plastic jars (19 × 33 cm) from a dairy facility situated in Al-Washlah, Riyadh, Saudi Arabia (24.39°N, 46.66°E). In the laboratory, the collected adults were transferred into a transparent cage (40 × 40 cm) and reared following the protocol of Abbas and Hafez [9]. Cotton wicks (3 cm) soaked in a 1:1 (by weight) solution of powdered milk and sugar in deionized water placed in plastic petri dishes (9 cm diameter) were provided for feeding of the adults, and these were refreshed every two days. The diet for the larvae consisted of wheat bran, yeast, dry milk powder, and sugar in the ratio 20:5:1.5:1.5 (g) made into a paste with 70 ml deionized water, provided in 500-ml plastic cups sited in the cages, for egg-laying and feeding. Each day, plastic cups in which eggs had been laid were removed from the cages and covered tightly with cloth to prevent hatched larvae from escaping. Larvae were transferred into glass beakers with fresh larval food after they had consumed the previous diet. Larvae were pupated in the glass beakers and the emerging adults were transferred into cages to form the next generation. Insects were maintained at 27°C ± 2°C, 65% ± 5% humidity, and under a 12h:12h (L/D) photoperiod in the laboratory.

The aforementioned field population of *M*. *domestica* (generation one; G_1_) was divided into two parts. One part, designated the susceptible strain, was cultured in the laboratory for 24 generations without exposure to any insecticide. The other part was selected by exposure to diflubenzuron for 20 generations to produce a diflubenzuron-resistant strain, designated Diflu-SEL. The Diflu-SEL generations G_5_ to G_24_ were screened with different concentrations of diflubenzuron (Table 2), the concentrations being chosen on the basis of larval survival, in order to obtain sufficient adults for the next generation. In 2000 ml glass beaker, two thousand 2^nd^ instar larvae were screened with diflubenzuron by the diet incorporation method in each generation [9]. The surviving larvae were allowed to pupate in glass beakers (Table 2). After emergence, the flies were moved to clean cages for the next generation and were maintained in the laboratory under the aforementioned conditions.

**Table 2:** History of selection with diflubenzuron to develop Diflu-SEL strain of *M*. *domestica*.

Generation	Concentration (ppm)	Number of exposed larvae	Number of emerged adults	Survival (%)
G_5_	0.86	2000	279	14
G_6_	0.86	2000	374	19
G_7_	0.86	2000	628	31
G_8_	0.86	2000	761	38
G_9_	0.86	2000	769	38
G_10_	0.86	2000	779	39
G_11_	0.86	2000	807	40
G_12_	0.86	2000	925	46
G_13_	0.86	2000	1290	65
G_14_	1.72	2000	789	39
G_15_	1.72	2000	859	43
G_16_	1.72	2000	945	47
G_17_	1.72	2000	1052	53
G_18_	3.44	2000	1509	75
G_19_	3.44	2000	1084	54
G_20_	3.44	2000	1135	57
G_21_	3.44	2000	1256	63
G_22_	3.44	2000	1348	67
G_23_	3.44	2000	1508	75
G_24_	3.44	2000	1591	80
Mean			984	49

Selection was not performed from G_1_–G_4_.

### Bioassays of *M*. *domestica* larvae

The toxicities of the IGRs against *M*. *domestica* larvae were assessed by diet incorporation bioassay as described by Abbas and Hafez [9]. Five serial concentrations (giving mortality range >0% to <100%) of each IGR were mixed into the larval food (formulated as described above), with three replicates for each bioassay. There were 10 2^nd^ instar larvae per replicate, 30 at each concentration, and 150 per bioassay. For the controls, the larval medium was made with deionized water only (3 replicates of 10 2^nd^ instar larvae each). All larval bioassays were performed under the abovementioned conditions. Mortality was noted at adult emergence; larvae failing to transform into adults were considered dead.

### Adult bioassays

The toxicities of the organophosphates and pyrethroids to adults flies of mixed sexes was evaluated by feeding as described by Abbas et al. [36]. Five concentrations (giving mortality range >0% to <100%) of each insecticide were made in twenty percent sucrose solution through serial dilution, with three replicates of each concentration for each bioassay. There were 10 adult flies in each replicate, 30 at each concentration, and 150 per bioassay. In the control, 30 adult flies were used (10 flies per replicate). The adult flies were transferred into plastic jars (11 × 15 cm) having perforations for aeration and with a cloth cover tied on to avoid escape of flies. Prior to treatment the flies were starved for 2 h. A cotton wick (~3 cm) was saturated with the treatment solution, placed in a 9-cm diameter petri dish, and the dish placed into the plastic jar to allow the flies to feed on the treatment solution. Cotton wicks saturated with twenty percent sugar solution only were provided to adult flies for control. All bioassays were performed under the abovementioned conditions. Mortality was noted after 48 h of exposure, after which LC_50_ values for the insecticides were calculated [36].

### Realized heritability (*h*^2^) values for diflubenzuron resistance

The *h*^2^ value for diflubenzuron resistance was calculated as described by Tabashnik [25] and Abbas et al. [24]:

$$h^{2}=R/S$$

where, *R* = selection response against diflubenzuron, and *S* = selection differential against diflubenzuron.

*R* was calculated using following formula:

$$R=\frac{\left[\log\;\left(final\;{LC}_{50}\;in\;Diflu-SEL\right)-\log\;\left(initial\;{LC}_{50}\;in\;field\;population\right)\right]}{n}$$

where *n* is the number of generations (G_5_–G_24_) screened with diflubenzuron.

*S* was determined as:

S=i×σp

where *i* = selection mortality, calculated according to the method of Tabashnik and McGaughey [37]:

$$i=1.583-0.0193336p+0.0000428p^{2}+3.65194/p.$$

where *p* = survival percentage of Diflu-SEL (G_5_–G_24_) screened with diflubenzuron.

The term *σp* was calculated as:

$$\sigma p=\frac{1}{average\;slope\;(G5-G24)}.$$

The number of generations (G) needed to produce a tenfold increase in LC_50_ was determined as previously described [24], using the equation:

$$G=1/h^{2}S.$$

The influence of the variables (slope and *h*^2^) on the projected rate of diflubenzuron resistance between G and selection mortality was assessed at calculated and assumed values of slope and *h*^2^.

### Bioassay data analyses

Bioassay data were analyzed by probit analyses using POLO Plus Software [38] to calculate the median lethal concentration (LC_50_), their fiducial limits (FLs), chi-squared (χ^2^), and slopes with their standard errors (±SEs). LC_50_ values with non-overlapped 95% FLs were considered significantly different [39]. Resistance levels (RR) were calculated as:

$$\frac{{LC}_{50}\;of\;an\;insecticide\;in\;the\;Diflu-SEL\;M.domestica}{{LC}_{50}\;of\;an\;insecticide\;in\;the\;susceptible\;or\;field\;collected\;M.domestica}$$

Cross-resistance (CR) and RR values for fourteen insecticides and diflubenzuron resistance were classified as follows: >100 = very high; 51–100 = high; 21–50 = moderate; 11–20 = low; 2–10 = very low; and ≤1 = no [24,36].

## Results

### Diflubenzuron resistance selection

The mean survival of *M*. *domestica* larvae at different concentrations of diflubenzuron was 49% in the G_5_–G_24_ generations (Table 2). Following laboratory selection, RR for diflubenzuron increased to 30.33 by G_5_ rising to 182.33 by G_24_ compared to the susceptible strain (Table 3). The LC_50_ for diflubenzuron increased from 0.91 ppm (95% FL 0.70–1.17) for Diflu-SEL G_5_ to 5.47 ppm (95% FL 3.26–18.51) for Diflu-SEL G_24_ (Table 3).

**Table 3:** Development of resistance to diflubenzuron in *M*. *domestica*.

Strain (Generation)	N^a^	Concentrations	LC_50_ (95% FL)^b^	Slope ± SE	*χ* ^2^	RR^c^
Susceptible (G_24_)	180	0.015625–0.25	0.03 (0.01–0.04)	2.52 ± 0.58	0.55	1.00
Diflu-SEL (G_5_)	180	0.215–3.44	0.91 (0.70–1.17)	2.18 ± 0.32	1.01	30.33
Diflu-SEL (G_6_)	180	0.215–3.44	0.93 (0.61–1.39)	1.30 ± 0.27	1.34	31.00
Diflu-SEL (G_7_)	180	0.215–3.44	0.94 (0.64–1.34)	1.45 ± 0.27	1.48	31.33
Diflu-SEL (G_8_)	180	0.215–3.44	0.97 (0.74–1.30)	1.67 ± 0.24	3.39	32.33
Diflu-SEL (G_9_)	180	0.25–4	1.00 (0.70–1.43)	1.48 ± 0.27	0.56	33.33
Diflu-SEL (G_10_)	180	0.25–4	1.16 (0.74–2.08)	0.94 ± 0.22	0.41	38.67
Diflu-SEL (G_11_)	180	0.25–4	1.19 (0.86–1.71)	1.57 ± 0.28	0.64	39.67
Diflu-SEL (G_12_)	180	0.25–4	1.69 (0.71–3.02)	1.64 ± 0.50	0.18	56.33
Diflu-SEL (G_13_)	180	0.25–4	1.78 (1.07–2.45)	2.82 ± 0.76	2.34	59.33
Diflu-SEL (G_14_)	180	0.25–4	2.15 (1.20–9.23)	0.74 ± 0.22	0.22	71.67
Diflu-SEL (G_15_)	180	0.25–4	2.29 (1.64–3.76)	1.64 ± 0.30	1.36	76.33
Diflu-SEL (G_16_)	180	0.25–4	2.73 (1.47–11.34)	0.94 ± 0.28	0.66	91.00
Diflu-SEL (G_17_)	180	0.25–4	2.83 (1.84–5.28)	1.81 ± 0.51	0.87	94.33
Diflu-SEL (G_18_)	180	0.25–4	3.00 (2.01–6.13)	1.46 ± 0.30	0.35	100.00
Diflu-SEL (G_19_)	180	0.25–4	3.31 (2.20–7.04)	1.49 ± 0.31	1.00	110.33
Diflu-SEL (G_20_)	180	0.25–4	3.31 (2.33–5.99)	1.81 ± 0.35	0.62	110.33
Diflu-SEL (G_21_)	180	0.25–4	3.43 (2.14–9.20)	1.25 ± 0.29	2.49	114.33
Diflu-SEL (G_22_)	180	0.25–4	3.56 (2.41–7.25)	1.64 ± 0.33	0.76	118.67
Diflu-SEL (G_23_)	180	0.25–4	3.79 (2.26–12.21)	1.18 ± 0.29	0.94	126.33
Diflu-SEL (G_24_)	180	0.50–8	5.47 (3.26–18.51)	1.49 ± 0.36	1.60	182.33

Bioassays were not performed at G_2_, G_3_, and G_4._

^a.^ Number of tested larvae in each bioassay.

^b.^ Median lethal concentration (ppm) with fiducial limits.

^c.^ Resistance ratio (RR = LC_50_ for diflubenzuron with selected strain/LC_50_ for diflubenzuron with susceptible strain).

### Realized heritability (*h*^2^)

The estimated *h*^2^ value for diflubenzuron resistance was 0.08 for Diflu-SEL G_24_ (Table 4).

**Table 4:** Realized heritability (*h*^2^) for diflubenzuron resistance in the Diflu-SEL strain of *M*. *domestica*.

Insecticide	Initial LC_50_ (log) ppm	Final LC_50_ (log) ppm	^a^G	^b^ *R*	^*c*^ *p*	^d^ *i*	Average slope	^e^ *σp*	^f^ *S*	^g^ *h* ^2^
Diflubenzuron	0.86 (−0.07)	5.47 (0.74)	20	0.04	49	0.81	1.51	0.66	0.54	0.08

^a^ number of generations selected with diflubenzuron

^b^ selection response

^c^ average surviving insects in selection

^d^ intensity of selection

^e^ phenotypic variation

^f^ selection differential

^g^ realized heritability of diflubenzuron resistance.

### Projected rate of development of diflubenzuron resistance

**Fig 1:**
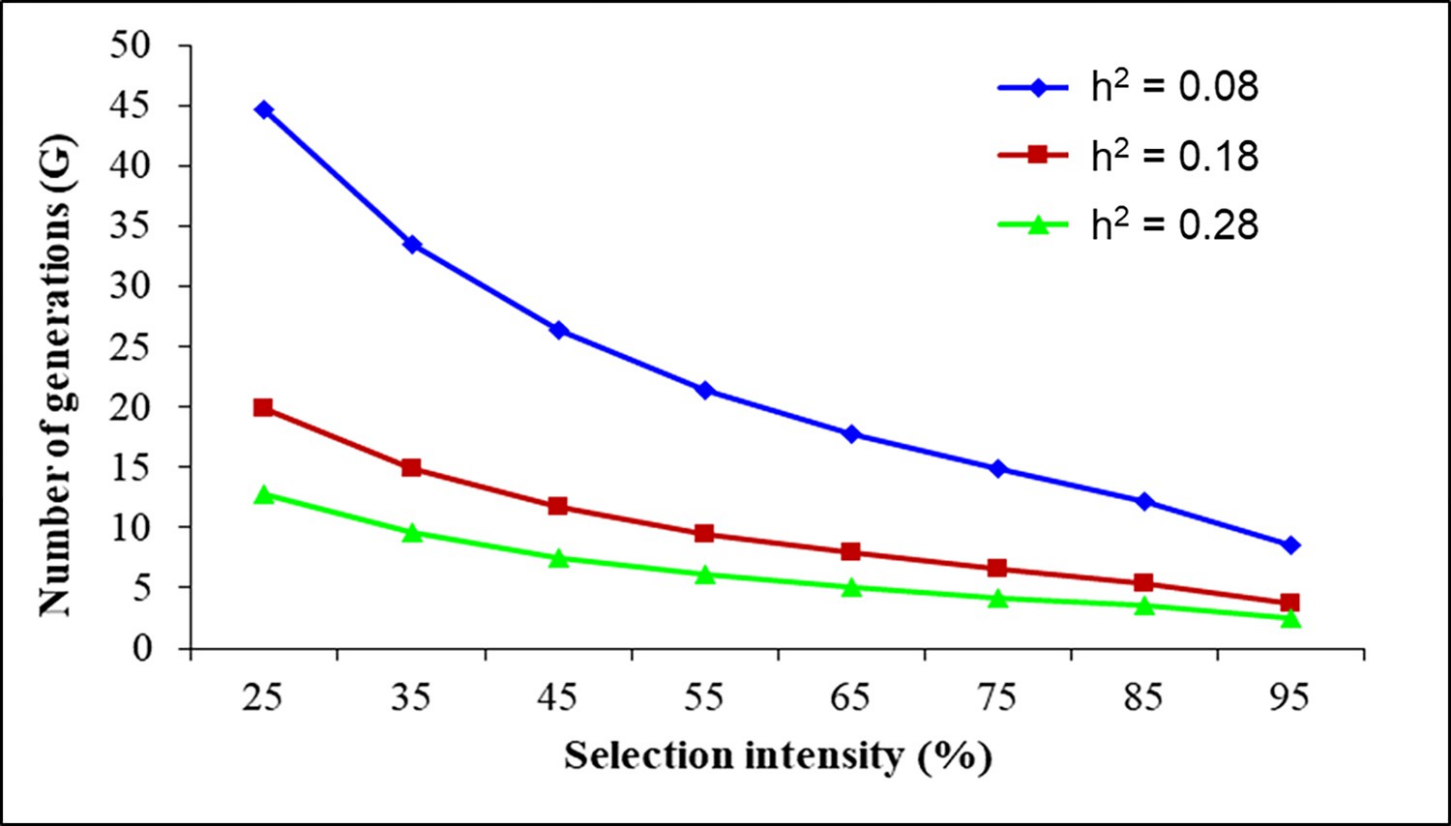
Effect of heritability on the number of generations of *M*. *domestica* needed for a 10-fold increase in LC_50_ for diflubenzuron at different selection intensities and constant slope (1.51).

**Fig 2:**
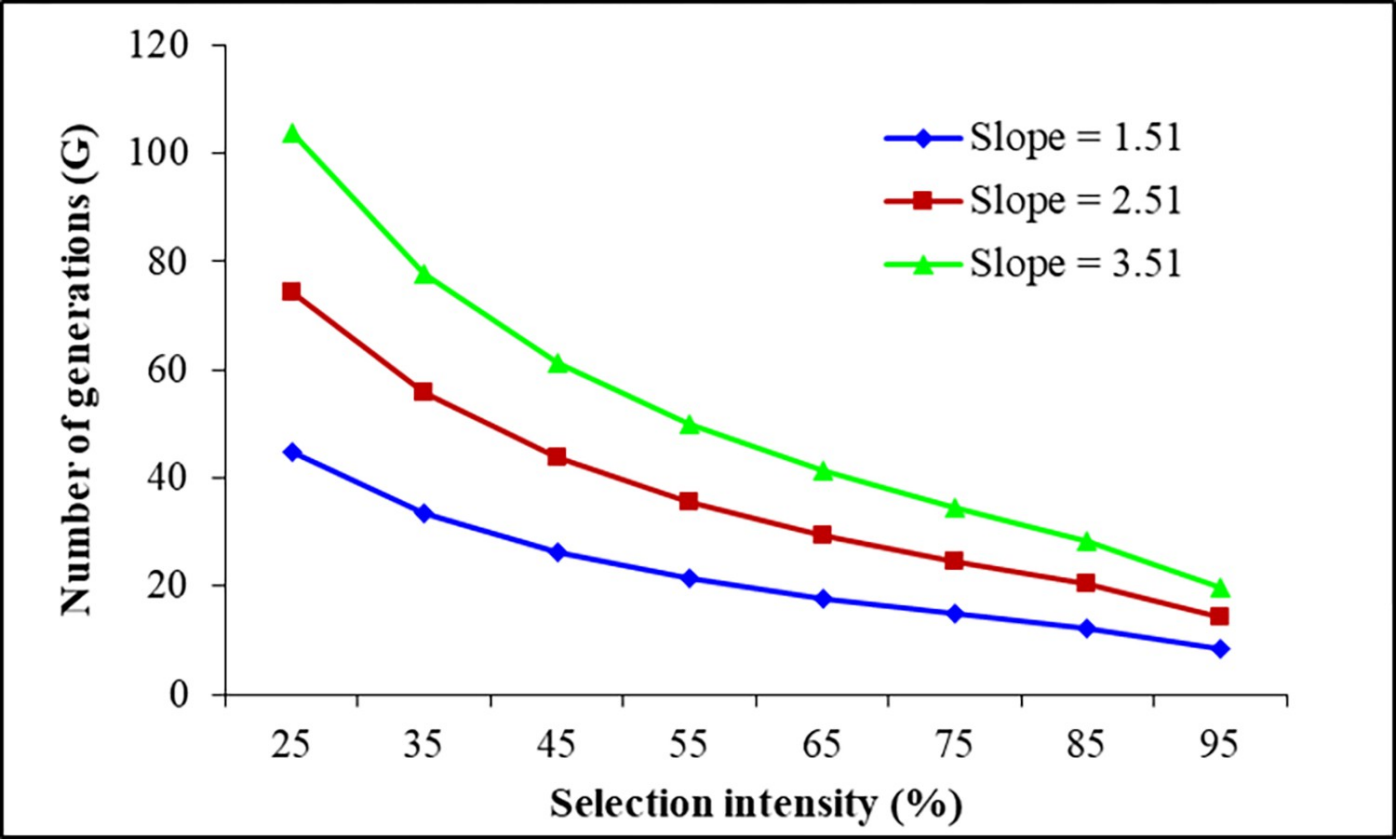
Effect of slope on the number of generations of *M*. *domestica* needed for a 10-fold increase in LC_50_ for diflubenzuron at different selection intensities and constant value of *h*^2^ (0.08).

Over a selection intensity range of 25% to 95%, the G values required for a tenfold increase in LC_50_ for diflubenzuron were 9–45, 4–20, and 2–13 at *h*^2^ values of 0.08, 0.18, and 0.28, respectively, with a constant slope of 1.51 (Fig 1). At a constant *h*^2^ value of 0.08 and with slopes of 1.51, 2.51, and 3.51, G values of 9–45, 14–74, and 20–104, respectively, equated to a tenfold increase in LC_50_ value in the Diflu-SEL *M*. *domestica* strain (Fig 2). These results indicate that changes in any of these variables can alter the rate of development of diflubenzuron resistance.

### Cross-resistance patterns

When compared to the field population, the Diflu-SEL *M*. *domestica* strain (G_24_) showed no CR between diflubenzuron and any of pyriproxyfen, methoxyfenozide, malathion, alpha-cypermethrin, bifenthrin, deltamethrin, cyfluthrin, or cypermethrin. Very low CR was exhibited between diflubenzuron and triflumuron, cyromazine, fenitrothion, and chlorpyrifos (Table 5).

**Table 5:** Cross-resistance to fourteen other insecticides in the diflubenzuron-selected strain of *M*. *domestica*.

Strain	Insecticide	N^a^	Concentrations	LC_50_ (95% FL)^b^	Slope ± SE	χ^2^	RR^c^	RR^d^
Susceptible (G_24_)	Triflumuron	180	0.125–2	0.21 (0.13–0.30)	1.69 ± 0.31	4.39		
	Pyriproxyfen	180	0.0078–0.125	0.01 (0.01–0.02)	2.00 ± 0.39	2.01		
	Methoxyfenozide	180	4–64	10.09 (7.57–12.90)	2.22 ± 0.33	0.67		
	Cyromazine	180	0.125–2	0.42 (0.33–0.53)	2.31 ± 0.33	0.51		
	Fenitrothion	180	128–2048	140.14 (22.77–264.23)	0.89 ± 0.26	0. 16		
	Malathion	180	128–2048	213.78 (62.52–368.78)	0.92 ± 0.26	0.22		
	Diazinon	180	2–32	2.99 (1.85–4.08)	1.87 ± 0.34	1.92		
	Pirimiphos-methyl	180	128–2048	153.83 (68.41–235.95)	1.41 ± 0.30	0.29		
	Chlorpyrifos	180	32–512	42.30 (18.61–65.30)	1.32 ± 0.29	4.87		
	Alpha-cypermethrin	180	16–256	22.21 (8.98–35.14)	1.20 ± 0.28	0.90		
	Bifenthrin	180	128–2048	254.76 (84.93–435.68)	0.90 ± 0.26	0.03		
	Deltamethrin	180	128–2048	146.51 (45.03–247.29)	1.12 ± 0.28	0.99		
	Cyfluthrin	180	128–2048	139.34 (36.91–243.39)	1.06 ± 0.27	0.54		
	Cypermethrin	180	128–2048	172.64 (59.97–283.84)	1.10 ± 0.27	0.32		
Field (G_1_)	Triflumuron*	240	0.0625–1	0.27 (0.08–0.41)	1.67 ± 0.38	0.84	1.29	
	Pyriproxyfen*	240	0.0625–1	0.22 (0.10–0.53)	0.80 ± 0.24	1.00	22.00	
	Methoxyfenozide*	240	4–64	20.79 (16.75–26.30)	2.15 ± 0.27	1.57	2.06	
	Cyromazine*	240	0.03125–0.5	0.22 (0.13–0.40)	2.47 ± 0.32	6.88	0.52	
	Fenitrothion	180	128–2048	196.36 (98.56–291.72)	1.38 ± 0.29	1.75	1.40	
	Malathion	180	128–2048	636.08 (467.39–897.35)	1.67 ± 0.28	0.28	2.98	
	Chlorpyrifos	180	128–2048	85.24 (26.93–138.14)	1.80 ± 0.44	0.28	2.02	
	Alpha-cypermethrin	180	16–256	72.35 (58. 42–90.05)	2.75 ± 0.37	0.32	3.26	
	Bifenthrin	180	128–2048	851.37 (498.93–2176.96)	0.90 ± 0.25	0.17	3.34	
	Deltamethrin	180	128–2048	764.10 (533.50–1216.99)	1.38 ± 0.27	1.20	5.22	
	Cyfluthrin	180	128–2048	526.85 (344.96–812.28)	1.25 ± 0.26	1.90	3.78	
	Cypermethrin	180	128–2048	329.49 (177.67–505. 97)	1.13 ± 0.26	0.25	1.91	
Diflu-SEL (G_24_)	Triflumuron	180	0.25–4	1.32 (0.77–2.52)	2.11 ± 0.32	4.20	6.29	4.89
	Pyriproxyfen	180	0.03125–0.5	0.07 (0.04–0.10)	1.46 ± 0.28	0.96	7.00	0.32
	Methoxyfenozide	180	4–64	16.81 (8.59–34.48)	1.89 ± 0.30	4.56	1.67	0.81
	Cyromazine	180	0.125–2	1.20 (0.96–1.59)	2.81 ± 0.46	2.74	2.86	5.45
	Fenitrothion	180	128–2048	493.10 (369.28–657.99)	1.87 ± 0.29	1.87	3.52	2.51
	Malathion	180	128–2048	791.60 (413.68–2705.87)	0.75 ± 0.25	1.20	3.70	1.24
	Diazinon	180	2–32	2.50 (1.26–3.69)	1.56 ± 0.32	0.29	0.84	-
	Pirimiphos-methyl	180	128–2048	181.74 (87.10–273.11)	1.38 ± 0.29	1.77	1.18	-
	Chlorpyrifos	180	128–2048	239.25 (153.22–326.87)	1.73 ± 0.30	1.13	5.66	2.81
	Alpha-cypermethrin	180	16–256	26.89 (12.92–40.86)	1.25 ± 0.28	0.83	1.21	0.37
	Bifenthrin	180	128–2048	529.29 (335.72–848.77)	1.16 ± 0.26	1.11	2.08	0.62
	Deltamethrin	180	128–2048	205.14 (106.85–302.01)	1.40 ± 0.29	1.49	1.40	0.27
	Cyfluthrin	180	128–2048	420.47 (212.72–730.14)	1.99 ± 0.30	4.09	3.02	0.80
	Cypermethrin	180	128–2048	358.62 (166.67–603.09)	0.94 ± 0.25	0.13	2.08	1.09

* Published data [9].

^a.^ Number of tested larvae in each bioassay.

^b.^ Median lethal concentration (ppm) with fiducial limits.

^c.^ Resistance ratio (LC_50_ for diflubenzuron in selected strain/LC_50_ for diflubenzuron in susceptible strain).

^d.^ Resistance ratio (LC_50_ for diflubenzuron in selected strain/LC_50_ for diflubenzuron in field population).

When compared with the susceptible strain, the Diflu-SEL *M*. *domestica* strain (G_24_) showed no CR between diflubenzuron and any of methoxyfenozide, diazinon, pirimiphos-methyl, alpha-cypermethrin, bifenthrin, deltamethrin, cyfluthrin, or cypermethrin. Very low CR was exhibited between diflubenzuron and triflumuron, pyriproxyfen, cyromazine, fenitrothion, malathion, or chlorpyrifos (Table 5).

## Discussion

Diflubenzuron, a chitin synthesis inhibitor, is commonly used alone or in combination with other insecticides to control various insect pests of medical importance, including *M*. *domestica*. Previously we have reported low to moderate resistance (RR = 9.33 to 28.67) to diflubenzuron in different populations of *M*. *domestica* [9]. In this study, selection of *M*. *domestica* with diflubenzuron over twenty generations increased resistance dramatically (RR = 182.33) in comparison to the susceptible strain. This suggests that *M*. *domestica* can rapidly developed a high level of diflubenzuron resistance under laboratory conditions. In agreement with our findings, *M*. *domestica* has been shown to rapidly develop high resistance to many insecticides under laboratory conditions, for example to imidacloprid (RR = 106) [40], spirotetramat (RR = 109) [41], pyriproxyfen (RR = 206) [33], cyromazine (RR = 211) [30], fipronil (RR = 430) [3], lambda-cyhalothrin (RR = 445) [27], chlorantraniliprole (RR = 750) [42], clothianidin (RR = 3827) [43], and methoxyfenozide (RR = 5254) [44]. This study conclusively shows that diflubenzuron resistance can likewise increase in *M*. *domestica*. The likely reason may be the existence of resistance allele(s) in the *M*. *domestica* population collected in the field [9]. However, further biochemical and molecular studies are required to explore the correlated phenomena.

Realized heritability (*h*^2^) values provide evidence of the risk of development of insecticide resistance in laboratory-selected strains of any pest [25,45]. In this study, the low estimated *h*^2^ value of 0.08 indicates low genetic variation and high phenotypic variation with lower tendency of *M*. *domestica* to develop diflubenzuron resistance genetically. This result is in agreement with those of other studies showing low values of *h*^2^ for insecticide resistance in *M*. *domestica*: 0.05 for fipronil [3], 0.06 for lambda-cyhalothrin [27], 0.17 for methoxyfenozide [28], 0.03 for pyriproxyfen [23], and 0.02 for flonicamid [24]. However, in contrast to our results, high values of *h*^2^ have been reported in insecticide-resistant *M*. *domestica*: 0.59 for spiromesifen [41], 0.32 for chlorantraniliprole [42], and 0.38 for clothianidin [43]. While field environmental conditions are varied compared to laboratory-controlled conditions [37,46], calculated values of *h*^2^ for diflubenzuron resistance by experimental selection in the laboratory have practical application for the control of *M*. *domestica*.

Assessment of insecticide resistance risk is an important step toward establishing rational and scientific resistance management strategies [3,24]. Estimation of the development of resistance (through G = 1/*h*^2^S) provides valuable insights into the risk of increased insecticide resistance in insect pests and for developing strategies to delay the problem [25,27,47,48]. The risk of development of resistance to fipronil, pyriproxyfen, spiromesifen, lambda-cyhalothrin, chlorantraniliprole, methoxyfenozide, clothianidin, and flonicamid have been reported previously in insecticide-induced resistant *M*. *domestica* [3,23,27,28,41–43]. Our results indicate that G values of 9–45, 4–20, and 2–13 would be needed to produce tenfold increases in LC_50_ for diflubenzuron at *h*^2^ values of 0.08, 0.18, and 0.28, respectively, with 25% to 95% selection mortality and a constant slope value of 1.51. G values of 9–45, 14–74, and 20–104 equate to slopes of 1.51, 2.51, and 3.51, respectively, at a constant *h*^2^ value of 0.08. These results show that with an increase in *h*^2^ for diflubenzuron resistance, the risk of developing resistance increases. Therefore, prudence is required when considering the risk of development of resistance to diflubenzuron when taking measures to control *M*. *domestica*.

The existence of CR in an insect pest affects the efficacy of insecticides that have never been used against that pest [24,46]. Therefore, knowledge of CR is useful when choosing effective insecticides for rotational use in a managed program [1,32,46,47,49]. In the present study, the Diflu-SEL strain of *M*. *domestica* showed no or very low CR between diflubenzuron and any of the tested insecticides in comparison with the susceptible or field strains. Some CR to triflumuron was expected because of its similar mode to diflubenzuron, but CR to the other tested insecticides was not expected as their modes of action differed [50]. However, a diflubenzuron-resistant strain of *M*. *domestica* from Denmark was shown to exhibit a very high CR to triflumuron (RR = 1000) [17]. A cyromazine-selected strain of *M*. *domestica* showed no CR to diflubenzuron, pyriproxyfen, or methoxyfenozide [30]. Similarly, spinosad- and s-methoprene-resistant strains of *Culex quinquefasciatus* Say were shown to exhibit no CR with diflubenzuron and pyriproxyfen [51,52]. A diflubenzuron-resistant strain of *Spodoptera littoralis* (Boisd.) showed no CR to two juvenoids (methoprene and triprene), but differing yet significant levels of CR to organochlorine, organophosphate, carbamate, and pyrethroid insecticides [53]. In Saudi Arabia, then, the absence of or very low CR between diflubenzuron and triflumuron (or the other tested insecticides) offers the option of alternation with diflubenzuron for the elimination of *M*. *domestica*.

It would be advisable for resistance management programs to be established for diflubenzuron to lengthen its potency against *M*. *domestica* in Saudi Arabia. Resistance should be monitored regularly to monitor its effectiveness for controlling *M*. *domestica*. Moreover, biological and cultural control measures should be adopted as elements of integrated pest management to reduce the usage of this insecticide. The low *h*^2^ value seen in this study provides encouragement for the management of diflubenzuron resistance. The absence of or very low CR between diflubenzuron and triflumuron, cyromazine, pyriproxyfen, methoxyfenozide, fenitrothion, chlorpyrifos, malathion, alpha-cypermethrin, bifenthrin, deltamethrin, cyfluthrin, or cypermethrin provides the opportunity for rotational usage of these insecticides to limit potential resistance in *M*. *domestica*, reducing insecticide-induced environmental damage.

## Supporting information

S1 File Bioassay data of different insecticides for susceptible strain of *M*. *domestica*. (PDF)

S2 File Bioassay data of different insecticides for field population of *M*. domestica. (PDF)

S3 File Bioassay data of different insecticides for diflubenzuron selected strain of *M*. domestica. (PDF)

S4 File Projected rate of resistance. (PDF)

S5 File Realized heritability. (PDF)
