# Supplementary material for: Risk assessment of resistance to diflubenzuron in Musca domestica: Realized heritability and cross-resistance to fourteen insecticides from different classes
Source: PLoS One. 2022 May 13;17(5):e0268261. doi: 10.1371/journal.pone.0268261 (PMC9106163; doi:10.1371/journal.pone.0268261)
Supplement: S1 File — (PDF) [file pone.0268261.s001.pdf]

S1. Bioassay data of different insecticides for susceptible strain (G24) of *M. domestica*

Deltamethrin			Fenitrothion			Cyromazine		
Concentrations (ppm)	NE	ND	Concentrations (ppm)	NE	ND	Concentrations (ppm)	NE	ND
2048	30	27	2048	30	26.00	2	30	28
1024	30	26	1024	30	23.00	1	30	25
512	30	20	512	30	21.00	0.5	30	16
256	30	18	256	30	19.00	0.25	30	10
128	30	15	128	30	14.00	0.125	30	3
Control	30	0	Control	30	0.00	Control	30	0
**Alpha-cypermethrin**			**Chlorpyrifos**			**Triflumuron**		
Concentrations (ppm)	NE	ND	Concentrations (ppm)	NE	ND	Concentrations (ppm)	NE	ND
256	30	28	512	30	30.00	2	30	30
128	30	24	256	30	23.00	1	30	26
64	30	20	128	30	21.00	0.5	30	19
32	30	17	64	30	17.00	0.25	30	15
16	30	14	32	30	15.00	0.125	30	13
Control	30	0	Control	30	0.00	Control	30	0
**Bifenthrin**			**Malathion**			**Methoxyfenozide**		
Concentrations (ppm)	NE	ND	Concentrations (ppm)	NE	ND	Concentrations (ppm)	NE	ND
2048	30	24	2048	30	24.00	64	30	29
1024	30	21	1024	30	22.00	32	30	26
512	30	18	512	30	20.00	16	30	19
256	30	15	256	30	16.00	8	30	14
128	30	12	128	30	12.00	4	30	5
Control	30	0	Control	30	0.00	Control	30	0
**Cypermethrin**			**Pirimiphos-methyl**			**Pyriproxyfen**		
Concentrations (ppm)	NE	ND	Concentrations (ppm)	NE	ND	Concentrations (ppm)	NE	ND
2048	30	27	2048	30	28.00	0.125	30	30
1024	30	24	1024	30	27.00	0.0625	30	28
512	30	20	512	30	23.00	0.03125	30	22
256	30	17	256	30	18.00	0.015625	30	17
128	30	14	128	30	14.00	0.0078125	30	15
Control	30	0	Control	30	0.00	Control	30	0
**Cyfluthrin**			**Diazinon**					
Concentrations (ppm)	NE	ND	Concentrations (ppm)	NE	ND			
2048	30	26	32	30	30.00			
1024	30	25	16	30	27.00			
512	30	23	8	30	23.00			
256	30	18	4	30	16.00			
128	30	14	2	30	13.00			
Control	30	0	Control	30	0.00			

NE = Number of exposed larvae or adults
ND = Number of dead larvae or adults
