# Supplementary material for: Risk assessment of resistance to diflubenzuron in Musca domestica: Realized heritability and cross-resistance to fourteen insecticides from different classes
Source: PLoS One. 2022 May 13;17(5):e0268261. doi: 10.1371/journal.pone.0268261 (PMC9106163; doi:10.1371/journal.pone.0268261)
Supplement: S2 File — (PDF) [file pone.0268261.s002.pdf]

S2. Bioassay data of different insecticides for field population (G1) of *M. domestica*

Deltamethrin			Fenitrothion		
Concentrations (ppm)	NE	ND	Concentrations (ppm)	NE	ND
2048	30	23	2048	30	29
1024	30	16	1024	30	24
512	30	12	512	30	20
256	30	7	256	30	17
128	30	5	128	30	13
Control	30	0	Control	30	0
**Alpha-cypermethrin**			**Chlorpyrifos**		
Concentrations (ppm)	NE	ND	Concentrations (ppm)	NE	ND
256	30	28	2048	30	30
128	30	22	1024	30	29
64	30	15	512	30	28
32	30	5	256	30	24
16	30	1	128	30	19
Control	30	0	Control	30	0
**Bifenthrin**			**Malathion**		
Concentrations (ppm)	NE	ND	Concentrations (ppm)	NE	ND
2048	30	20	2048	30	25
1024	30	16	1024	30	19
512	30	13	512	30	13
256	30	9	256	30	9
128	30	8	128	30	4
Control	30	0	Control	30	0
**Cypermethrin**					
Concentrations (ppm)	NE	ND			
2048	30	25			
1024	30	22			
512	30	17			
256	30	14			
128	30	10			
Control	30	0			
**Cyfluthrin**					
Concentrations (ppm)	NE	ND			
2048	30	25			
1024	30	17			
512	30	15			
256	30	10			
128	30	8			
Control	30	0			

NE = Number of exposed larvae or adults

ND = Number of dead larvae or adults
