# Supplementary material for: Risk assessment of resistance to diflubenzuron in Musca domestica: Realized heritability and cross-resistance to fourteen insecticides from different classes
Source: PLoS One. 2022 May 13;17(5):e0268261. doi: 10.1371/journal.pone.0268261 (PMC9106163; doi:10.1371/journal.pone.0268261)
Supplement: S3 File — (PDF) [file pone.0268261.s003.pdf]

S3. Bioassay data of different insecticides for diflubenzuron selected strain (G24) of *M. domestica*

**Deltamethrin**			**Fenitrothion**			**Cyromazine**		
Concentrations (ppm)	NE	ND	Concentrations (ppm)	NE	ND	Concentrations (ppm)	NE	ND
2048	30	28	2048	30	28	2	30	20
1024	30	26	1024	30	20	1	30	15
512	30	19	512	30	14	0.5	30	5
256	30	16	256	30	9	0.25	30	0
128	30	13	128	30	5	0.125	30	0
Control	30	0	Control	30	0	Control	30	0
**Alpha-cypermethrin**			**Chlorpyrifos**			**Triflumuron**		
Concentrations (ppm)	NE	ND	Concentrations (ppm)	NE	ND	Concentrations (ppm)	NE	ND
256	30	27	2048	30	28	4	30	24
128	30	23	1024	30	27	2	30	19
64	30	22	512	30	20	1	30	15
32	30	15	256	30	17	0.5	30	7
16	30	12	128	30	9	0.25	30	0
Control	30	0	Control	30	0	Control	30	0
**Bifenthrin**			**Malathion**			**Methoxyfenozide**		
Concentrations (ppm)	NE	ND	Concentrations (ppm)	NE	ND	Concentrations (ppm)	NE	ND
2048	30	24	2048	30	17	64	30	28
1024	30	17	1024	30	17	32	30	17
512	30	15	512	30	15	16	30	15
256	30	10	256	30	11	8	30	10
128	30	8	128	30	7	4	30	3
Control	30	0	Control	30	0	Control	30	0
**Cypermethrin**			**Pirimiphos-methyl**			**Pyriproxyfen**		
Concentrations (ppm)	NE	ND	Concentrations (ppm)	NE	ND	Concentrations (ppm)	NE	ND
2048	30	23	2048	30	29	0.5	30	26
1024	30	20	1024	30	25	0.25	30	24
512	30	16	512	30	20	0.125	30	21
256	30	14	256	30	17	0.0625	30	15
128	30	10	128	30	14	0.03125	30	8
Control	30	0	Control	30	0	Control	30	0
**Cyfluthrin**			**Diazinon**					
Concentrations (ppm)	NE	ND	Concentrations (ppm)	NE	ND			
2048	30	26	32	30	29			
1024	30	24	16	30	27			
512	30	18	8	30	23			
256	30	13	4	30	18			
128	30	2	2	30	14			
Control	30	0	Control	30	0			

NE = Number of exposed larvae or adults

ND = Number of dead larvae or adults
