# Supplementary material for: Risk assessment of resistance to diflubenzuron in Musca domestica: Realized heritability and cross-resistance to fourteen insecticides from different classes
Source: PLoS One. 2022 May 13;17(5):e0268261. doi: 10.1371/journal.pone.0268261 (PMC9106163; doi:10.1371/journal.pone.0268261)
Supplement: S4 File — (PDF) [file pone.0268261.s004.pdf]

Diflubenzuron **Heritability 0.08**

Survival % p	p2	i	Mean slope	σp	S	h2	H2S	G	Selection intensity (Mortality)
75	5625	0.42	1.51	0.66	0.28	0.08	0.02	45	25
65	4225	0.56	1.51	0.66	0.37	0.08	0.03	34	35
55	3025	0.72	1.51	0.66	0.47	0.08	0.04	26	45
45	2025	0.88	1.51	0.66	0.58	0.08	0.05	21	55
35	1225	1.06	1.51	0.66	0.70	0.08	0.06	18	65
25	625	1.27	1.51	0.66	0.84	0.08	0.07	15	75
15	225	1.55	1.51	0.66	1.02	0.08	0.08	12	85
5	25	2.22	1.51	0.66	1.47	0.08	0.12	9	95

Diflubenzuron **Heritability 0.18**

Survival % p	p2	i	Mean slope	σp	S	h2	H2S	G
75	5625	0.42	1.51	0.66	0.28	0.18	0.05	20
65	4225	0.56	1.51	0.66	0.37	0.18	0.07	15
55	3025	0.72	1.51	0.66	0.47	0.18	0.09	12
45	2025	0.88	1.51	0.66	0.58	0.18	0.10	10
35	1225	1.06	1.51	0.66	0.70	0.18	0.13	8
25	625	1.27	1.51	0.66	0.84	0.18	0.15	7
15	225	1.55	1.51	0.66	1.02	0.18	0.18	5
5	25	2.22	1.51	0.66	1.47	0.18	0.26	4

Diflubenzuron **Heritability 0.28**

Survival % p	p2	i	Mean slope	σp	S	h2	H2S	G
75	5625	0.42	1.51	0.66	0.28	0.28	0.08	13
65	4225	0.56	1.51	0.66	0.37	0.28	0.10	10
55	3025	0.72	1.51	0.66	0.47	0.28	0.13	8
45	2025	0.88	1.51	0.66	0.58	0.28	0.16	6
35	1225	1.06	1.51	0.66	0.70	0.28	0.20	5
25	625	1.27	1.51	0.66	0.84	0.28	0.24	4
15	225	1.55	1.51	0.66	1.02	0.28	0.29	3
5	25	2.22	1.51	0.66	1.47	0.28	0.41	2

Diflubenzuron **Slope 1.51**

p	p2	i	Mean slope	σp	S	h2	H2S	G
75	5625	0.42	1.51	0.66	0.28	0.08	0.02	45
65	4225	0.56	1.51	0.66	0.37	0.08	0.03	34
55	3025	0.72	1.51	0.66	0.47	0.08	0.04	26
45	2025	0.88	1.51	0.66	0.58	0.08	0.05	21
35	1225	1.06	1.51	0.66	0.70	0.08	0.06	18
25	625	1.27	1.51	0.66	0.84	0.08	0.07	15
15	225	1.55	1.51	0.66	1.02	0.08	0.08	12
5	25	2.22	1.51	0.66	1.47	0.08	0.12	9

Diflubenzuron **Slope 2.51**

p	p2	i	Mean slope	σp	S	h2	H2S	G
75	5625	0.42	2.51	0.40	0.17	0.08	0.01	74
65	4225	0.56	2.51	0.40	0.22	0.08	0.02	56
55	3025	0.72	2.51	0.40	0.29	0.08	0.02	44
45	2025	0.88	2.51	0.40	0.35	0.08	0.03	36
35	1225	1.06	2.51	0.40	0.42	0.08	0.03	30
25	625	1.27	2.51	0.40	0.51	0.08	0.04	25
15	225	1.55	2.51	0.40	0.62	0.08	0.05	20
5	25	2.22	2.51	0.40	0.88	0.08	0.07	14

Diflubenzuron **Slope 3.51**

p	p2	i	Mean slope	σp	S	h2	H2S	G
75	5625	0.42	3.51	0.28	0.12	0.08	0.01	104
65	4225	0.56	3.51	0.28	0.16	0.08	0.01	78
55	3025	0.72	3.51	0.28	0.20	0.08	0.02	61
45	2025	0.88	3.51	0.28	0.25	0.08	0.02	50
35	1225	1.06	3.51	0.28	0.30	0.08	0.02	41
25	625	1.27	3.51	0.28	0.36	0.08	0.03	34
15	225	1.55	3.51	0.28	0.44	0.08	0.04	28
5	25	2.22	3.51	0.28	0.63	0.08	0.05	20
