# Supplementary material for: Risk assessment of resistance to diflubenzuron in Musca domestica: Realized heritability and cross-resistance to fourteen insecticides from different classes
Source: PLoS One. 2022 May 13;17(5):e0268261. doi: 10.1371/journal.pone.0268261 (PMC9106163; doi:10.1371/journal.pone.0268261)
Supplement: S5 File — (PDF) [file pone.0268261.s005.pdf]

Estimation of realized heritability

$i = 1.583 – 0.0193336p + 0.0000428p2 + 3.65194/p$

Insecticide	Initial LC50	Log (Initial LC50)	Final LC50	log (Final LC50)	log (final LC50-initial LC50)	number of generations (n)	R = log (final LC50 - initial LC50)/n	p (Av. Percentage of survival rate)	P2	i	Mean slope	σp	$S = i * \sigma p$	$h^{2 = R / S}$
Diflubenzuro	0.86	-0.07	5.47	0.74	0.80	20	0.0402	49	2401	0.81	1.51	0.66	0.54	0.075
